# The RNA-binding protein ELAV regulates Hox RNA processing, expression and function within the *Drosophila* nervous system

**DOI:** 10.1242/dev.101519

**Published:** 2014-05

**Authors:** Ana Rogulja-Ortmann, Joao Picao-Osorio, Casandra Villava, Pedro Patraquim, Elvira Lafuente, Julie Aspden, Stefan Thomsen, Gerhard M. Technau, Claudio R. Alonso

**Affiliations:** 1 Institute of Genetics, University of Mainz, Mainz D-55099, Germany; 2 School of Life Sciences, University of Sussex, Brighton BN1 9QG, UK

**Keywords:** Central nervous system, *Drosophila*, ELAV/Hu, Hox, RNA-binding protein, RNA processing, Alternative polyadenylation (APA), Alternative splicing, Segment-specific apoptosis

## Abstract

The regulated head-to-tail expression of Hox genes provides a coordinate system for the activation of specific programmes of cell differentiation according to axial level. Recent work indicates that Hox expression can be regulated via RNA processing but the underlying mechanisms and biological significance of this form of regulation remain poorly understood. Here we explore these issues within the developing *Drosophila* central nervous system (CNS). We show that the pan-neural RNA-binding protein (RBP) ELAV (Hu antigen) regulates the RNA processing patterns of the Hox gene *Ultrabithorax* (*Ubx*) within the embryonic CNS. Using a combination of biochemical, genetic and imaging approaches we demonstrate that ELAV binds to discrete elements within *Ubx* RNAs and that its genetic removal reduces Ubx protein expression in the CNS leading to the respecification of cellular subroutines under *Ubx* control, thus defining for the first time a specific cellular role of ELAV within the developing CNS. Artificial provision of ELAV in glial cells (a cell type that lacks ELAV) promotes Ubx expression, suggesting that ELAV-dependent regulation might contribute to cell type-specific Hox expression patterns within the CNS. Finally, we note that expression of *abdominal A* and *Abdominal B* is reduced in *elav* mutant embryos, whereas other Hox genes (*Antennapedia*) are not affected. Based on these results and the evolutionary conservation of ELAV and Hox genes we propose that the modulation of Hox RNA processing by ELAV serves to adapt the morphogenesis of the CNS to axial level by regulating Hox expression and consequently activating local programmes of neural differentiation.

## INTRODUCTION

The nervous system of vertebrates and invertebrates shows a remarkable level of regionalisation along the anteroposterior (AP) axis, so that the identity, arrangement and connectivity of nerve cells change according to axial level (Arendt and Nubler-Jung, 1999; Lumsden and Keynes, 1989; Lumsden and Krumlauf, 1996; Reichert, 2002; Thor, 1995). At the molecular level, axial neural specification relies centrally on the regulated expression of the Hox genes, a group of evolutionarily conserved genes that encode a family of homeodomain-containing transcription factors that activate specific cell differentiation programmes in different parts of the nervous system (McGinnis and Krumlauf, 1992; Alonso, 2002; Mallo and Alonso, 2013). An understanding of the molecular mechanisms underlying Hox gene expression and function within the CNS is thus crucial to decipher the ability of these genes to direct pluripotent neural populations along different developmental routes.

Hox gene expression can be regulated at multiple levels through a variety of transcriptional and post-transcriptional mechanisms (Simon et al., 1990; Shimell et al., 1994; Alonso and Wilkins, 2005; Alonso, 2012; Mallo and Alonso, 2013). Previous work in our laboratory and elsewhere has demonstrated that, in *Drosophila*, several Hox transcripts undergo mRNA processing by alternative splicing and alternative polyadenylation (Akam and Martinez-Arias, 1985; Kornfeld et al., 1989; Lopez and Hogness, 1991; O'Connor et al., 1988; Reed et al., 2010; Thomsen et al., 2010), that these processes affect Hox gene expression and function in a range of developmental contexts (Mann and Hogness, 1990; Subramaniam et al., 1994; Thomsen et al., 2010; Reed et al., 2010; de Navas et al., 2011) and are evolutionarily conserved over large phylogenetic distances (Bomze and Lopez, 1994; Patraquim et al., 2011). Notably, within the *Drosophila* embryonic CNS several Hox genes express specific splicing and 3′UTR isoforms (Thomsen et al., 2010), suggesting that RNA processing might represent a control system involved in fine-grain regulation of Hox expression within the CNS. Nonetheless, the underlying mechanisms and biological relevance of Hox RNA processing within neural tissues remain largely unknown.

Here we apply a reverse genetics approach designed to detect factors involved in Hox RNA processing, focusing on genes expressed within the *Drosophila* embryonic CNS. As a gene model we use the Hox gene *Ultrabithorax* (*Ubx*), as this is the system in which RNA processing is understood in greatest detail (Hatton et al., 1998; Alonso and Akam, 2003; de la Mata et al., 2003; Reed et al., 2010; Alonso, 2012; Mallo and Alonso, 2013). Our approach led us to identify the pan-neural RNA-binding protein (RBP) ELAV (also known as Hu antigen) (Robinow et al., 1988; Robinow and White, 1988; Good, 1995) as a key regulator of *Ubx* RNA processing, expression and function within the *Drosophila* embryonic CNS and to define what is, to our knowledge, the first specific cellular role of ELAV during neural development. We discuss the implications of our findings for the understanding of the molecular programmes controlling neural differentiation along the head-to-tail axis of animals.

## RESULTS

### ELAV regulates *Ubx* RNA processing in the embryonic CNS

**Fig. 1.**
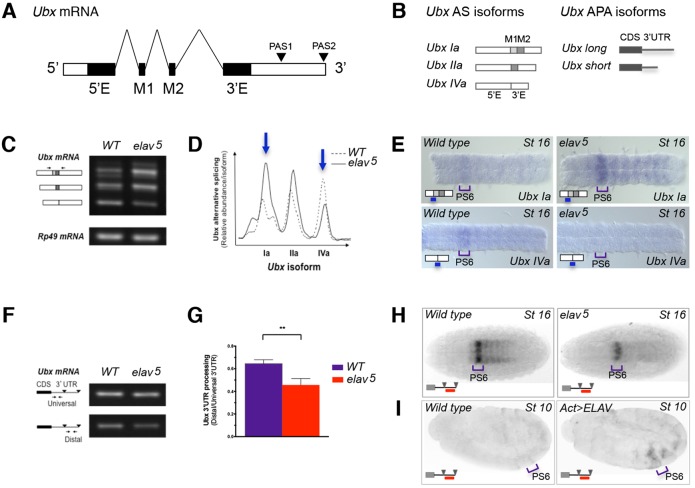
**The RNA-binding protein ELAV regulates Hox RNA processing in the *Drosophila* CNS.** (A,B) The *Drosophila Ubx* gene produces a spectrum of RNA isoforms via alternative splicing (AS) and alternative polyadenylation (APA). *Ubx* AS isoforms differ from each other by the presence/absence of small (micro) exons termed M1 and M2; *Ubx* APA leads to the formation of mRNAs bearing a long or short 3′UTR. PAS1, polyadenylation site 1; PAS2, polyadenylation site 2; 5′E, 5′ exon; 3′E, 3′ exon. 5′ and 3′ mRNA ends are indicated. (C,D) Molecular analysis of *Ubx* AS profiles in late *Drosophila* embryos reveals that changes in ELAV expression lead to a significant change in *Ubx* AS patterns, especially concerning *Ubx* isoforms Ia and IVa which are over-represented and under-represented, respectively (arrows), in *elav* mutant (*elav^5^*) embryos. (E) Developmental expression analysis of specific *Ubx* splicing isoforms in dissected embryonic ventral nerve cords (anterior is to the left) using oligo *in situ* hybridisation confirms that changes in ELAV expression lead to changes in the expression level of *Ubx* splicing isoforms in the developing CNS. PS6, parasegment 6. (F,G) Molecular analysis of *Ubx* APA patterns shows that changes in ELAV level lead to a significant change in the abundance of long and short 3′UTR isoforms at late embryogenesis (*n*=3). CDS, coding sequence. Error bars indicate s.e.m. **P<0.01 [Wilcoxon matched-pairs signed rank test (non-parametric *t*-test)]. (H,I) Mutation in the RBP ELAV leads to a reduction in the expression of long 3′UTR forms of *Ubx* within the CNS of embryos (H). Notably, ectopic expression of ELAV at the germband extension stage (I) shows a clear increase in the expression of long 3′UTR isoforms of *Ubx*, indicating that ELAV is sufficient to induce a change in *Ubx* RNA processing *in vivo*.

Embryos carrying a null mutation in the *elav* gene [*elav^5^* mutants (Yao et al., 1993)] produced patterns of *Ubx* alternative splicing (AS) and alternative polyadenylation (APA) that were significantly different from those found in wild-type embryos (Fig. 1A-H), indicating that *Drosophila* ELAV is necessary for normal *Ubx* RNA processing within the embryonic CNS. During normal embryonic development, alternatively spliced forms of *Ubx* are produced in a specific spatiotemporal pattern of expression: isoforms containing both M1 and M2 exons (*Ubx* I, Fig. 1A,B) account for most of the *Ubx* mRNAs expressed in epidermis and mesoderm, whereas isoforms lacking M1 and/or M2 (*Ubx* II and IV, Fig. 1A,B) are most abundant in neural cells (Artero et al., 1992). With regards to APA, *Ubx* mRNA forms bearing short 3′UTRs are dominant during early and mid-embryogenesis, but later on in development (late embryogenesis) the formation of long 3′UTR forms becomes predominant, especially in neural tissue (Akam and Martinez-Arias, 1985; Kornfeld et al., 1989; O'Connor et al., 1988; Lopez and Hogness, 1991; Thomsen et al., 2010).

We examined *Ubx* RNA processing profiles using two independent experimental approaches: RT-PCR and RNA *in situ* hybridisations in dissected ventral nerve cords (i.e. oligo *in situ* hybridisations) or in whole embryos (Fig. 1C-H). Remarkably, both approaches were consistent in showing that, in *elav^5^* mutants at stage 16, *Ubx* splicing isoforms Ia and IVa were over- and under-represented, respectively (Fig. 1C-E) (whereas isoform *Ubx* IIa showed comparatively little change across genotypes). This analysis also revealed an overall reduction in *Ubx* long 3′UTR mRNAs (Fig. 1F-H). Notably, we also observed that ectopic expression of ELAV during gastrulation is sufficient to change the pattern of *Ubx* APA by directing the formation of *Ubx* mRNA forms carrying long 3′UTRs that are typically observed only in the CNS (Fig. 1I) instead of the shorter 3′UTRs commonly expressed at this stage (Thomsen et al., 2010). This indicates that ELAV is both necessary and sufficient to reprogramme *Ubx* 3′UTR RNA processing during embryogenesis. At later stages (stage 16), when the majority of *Ubx* mRNAs normally exhibit a long 3′UTR (Thomsen et al., 2010), overexpression of ELAV causes no detectable change, suggesting that the system is likely to already be producing as much of the long *Ubx* 3′UTRs as it possibly can (supplementary material Fig. S2). Altogether, these experiments demonstrate that ELAV is necessary and sufficient to modify the RNA processing patterns of a *Drosophila* Hox gene.

### ELAV interacts with discrete elements within *Ubx* RNA

**Fig. 2.**
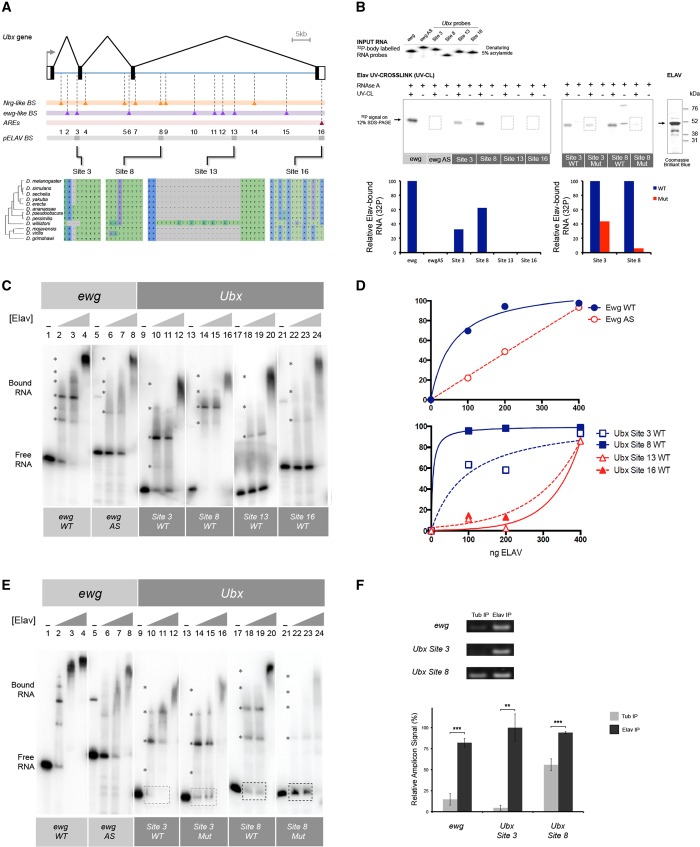
**ELAV binds to discrete elements within *Ubx* RNAs*.*** (A) Based on the computational detection of RNA sequence elements with high similarity to those present in other ELAV targets (*Neuroglian*, *Nrg*; *erect wing*, *ewg*) and AU-rich elements (AREs) we determined that the *Ubx* locus contains at least 16 putative binding sites for the neural protein ELAV. Phylogenetic analysis of *Ubx* sequences within distantly related *Drosophilids* reveals that a subset of ELAV putative binding sites (EBSs) (sites 3, 8, 13 and 16) have been conserved for more than 60 million years of independent evolution (i.e. ultraconserved), suggesting that they might be functionally relevant. (B) UV cross-linking experiments reveal that *Drosophila* ELAV is able to bind to discrete elements in *Ubx* mRNAs. A panel of radioactively labelled (^32^P) RNA probes (top, see Input RNA gel), including elements from the *ewg* mRNA (a previously described experimentally validated ELAV target) and the four ultraconserved *Ubx* ELAV binding sites detected bioinformatically (sites 3, 8, 13 and 16), were incubated with ELAV protein and treated with RNase A, with or without prior UV crosslinking (UV-CL; − or +) and the products of these reactions were resolved by PAGE-SDS. These experiments revealed that ELAV has high affinity for sites 3 and 8, but very low affinity for sites 13 and 16 (bottom left shows quantification of ELAV-bound RNA per *Ubx* mRNA site). Furthermore, site-specific mutagenesis affecting the core binding sequences of ELAV in sites 3 and 8 led to a significant reduction in interaction between ELAV and *Ubx* mRNAs (bottom right shows quantification of ELAV-bound RNA per wild-type or mutated *Ubx* mRNA site). These experiments show that ELAV is able to strongly interact with specific sites within *Ubx* mRNAs. AS, antisense. (C-E) EMSAs using ^32^P*-*labelled *Ubx* and *ewg* RNAs further confirms that ELAV has high affinity for *Ubx* sites 3 and 8 and that mutation of these sites leads to a change in ELAV-RNA interaction. (C) RNA probes were radioactively labelled and quantified so that equal molar units of each type of RNA were included. ELAV forms a range of complexes as a result of multimerisation on *ewg* probes (asterisks). Multimerisation is also observed on *Ubx* probes (asterisks). Note that the amount of probe is not saturating, therefore allowing us to estimate the affinity of ELAV protein for each RNA probe by following the disappearance of ‘free RNA’ signal as a function of the increase in ELAV protein concentration ([ELAV]) in the experiment: if free RNA probe signal disappears at lower concentrations of ELAV then this is an indication of higher affinity of ELAV for such RNA sequences as compared with those for which ELAV concentration has to reach maximum levels to generate shifted complexes. (D) Quantification of the binding profiles of ELAV protein to *ewg* and *Ubx* RNA probes as shown in C. (E) EMSA using mutated versions of probes 3 and 8 shows reduced binding activity (dashed rectangles) and a change in the range of protein-RNA complexes formed. (F) RNA cross-linking and immunoprecipitation (RNA-CLIP) assay using embryonic nuclear extracts shows that anti-ELAV antibodies are able to precipitate RNA derived from *ewg* and *Ubx* sites 3 and 8. Altogether, these experiments support the model that ELAV interacts directly with *Ubx* RNAs via sequences in the ultraconserved sites EBS3 and EBS8. Error bars indicate s.e.m. **P<0.01, ***P<0.001 [Wilcoxon matched-pairs signed rank test (non-parametric *t*-test)].

To determine the mechanisms that link ELAV expression level with *Ubx* RNA processing events we explored the model that ELAV exerts its effects via direct interaction with *Ubx* RNA. ELAV/Hu proteins possess an RNA-binding unit bearing three RNA recognition motifs that show high affinity for AU-rich elements (Wang and Tanaka, 2001). Given that *elav* encodes an RBP we scanned *Ubx* RNA sequences for putative ELAV binding sites (EBS). Bioinformatic analysis of the *Ubx* locus revealed the existence of several EBSs (Fig. 2A; supplementary material Fig. S3 and legend). Remarkably, 16 *Ubx* EBSs were significantly evolutionarily conserved across *Drosophila* species that evolved independently from each other for over 60 million years, suggesting a potential functional relevance of these sites (Fig. 2A).

To test the hypothesis that ELAV interacts with *Ubx* RNA in a direct fashion, we determined to what extent ELAV was able to interact with radioactively labelled *Ubx* RNAs *in vitro* (Fig. 2B-E). We used two distinct experimental approaches: protein/RNA UV crosslinking followed by RNase A treatment (Fig. 2B) and RNA electrophoretic mobility shift assay (EMSA) (Fig. 2C-E). In both biochemical approaches we tested ultraconserved EBSs (Fig. 2A) using sequences derived from the previously described ELAV target *erect wing* (*ewg*) as positive [*ewg* wild type (WT)] and negative [*ewg* antisense (AS)] controls (Koushika et al., 2000; Soller and White, 2003). Notably, analysis of those sites showing the highest level of evolutionary conservation (supplementary material Fig. S3) revealed, by means of these two independent experimental approaches, that ELAV strongly interacts with *Ubx* RNAs directly through high affinity binding to ultraconserved sites EBS3 and EBS8, but very weakly with sites EBS13 and EBS16. Furthermore, site-specific mutation of the predicted ELAV binding sequences contained within EBS3 and EBS8 (supplementary material Fig. S3) led to different patterns of ELAV binding to *Ubx* RNA (Fig. 2E): EMSA using mutated versions of probes 3 and 8 showed reduced binding activity and a change in the range of protein-RNA complexes formed. These experiments also reveal the formation of protein-RNA complexes, including multimerised forms of ELAV on RNA (Fig. 2C,D). This has been observed previously in the interaction of ELAV with *ewg* RNAs (Soller and White, 2005).

To determine the extent to which ELAV-*Ubx* RNA interactions take place within the physiological environment of the developing embryo we developed a series of RNA cross-linking and immunoprecipitation (RNA-CLIP) assays using embryonic nuclear extracts (Fig. 2F). These experiments confirmed that ELAV interacts with *Ubx* RNAs at EBS3 and EBS8 in normal tissue, while a negative control antibody (anti-Tubulin) rendered a significantly lower level of *Ubx* RNA precipitation (Fig. 2F). We conclude that our biochemical analysis supports the model that ELAV interacts with *Ubx* RNAs directly via sites in *Ubx* introns 1 and 3 (EBS3 and EBS8, respectively; see Fig. 2A).

### ELAV regulates Ubx expression levels within the CNS

**Fig. 3.**
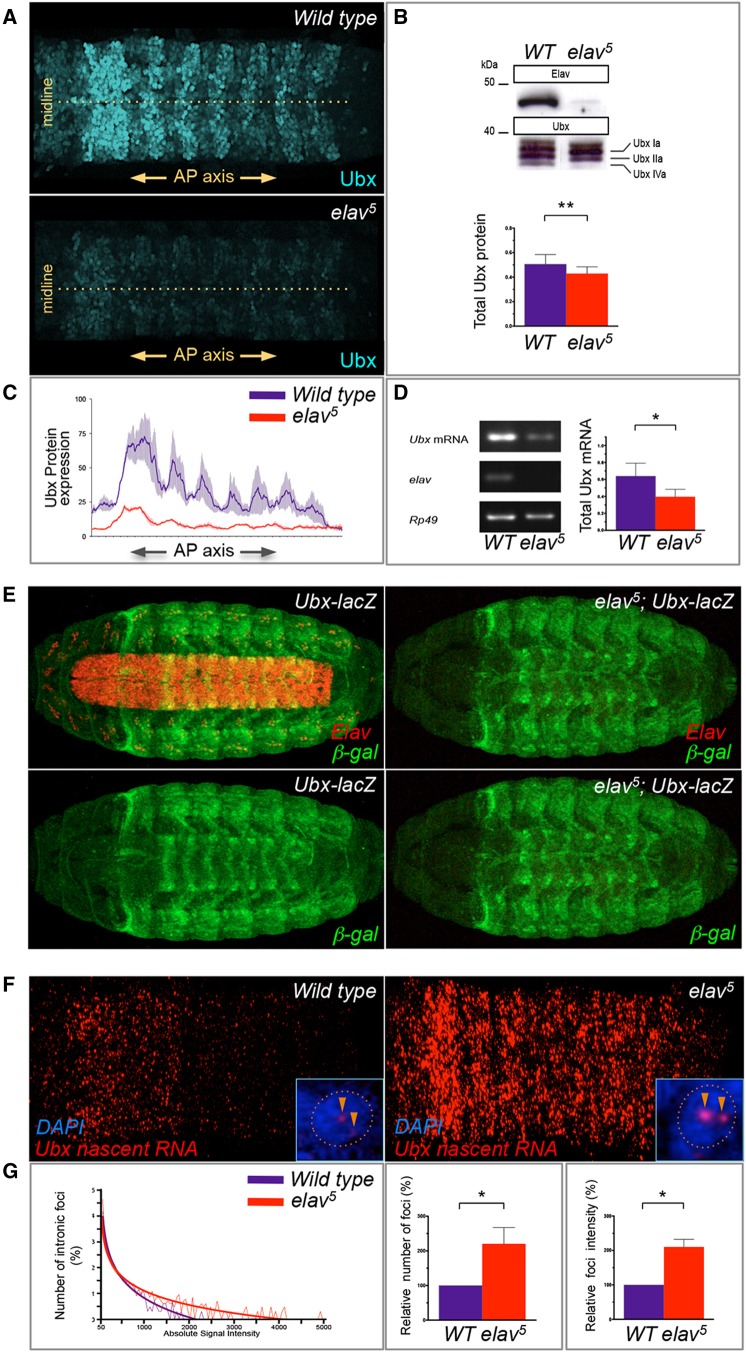
**ELAV removal leads to reduced expression of *Ubx* mRNA and protein and promotes accumulation of nascent *Ubx* RNA transcripts within the *Drosophila* CNS.** (A) Immunostaining of dissected embryonic ventral nerve cords (anterior is to the left) showing the expression of Ubx protein in wild-type and homozygous *elav* (*elav^5^*) mutant embryos at stage 16; *elav^5^* mutant embryos express significantly lower levels of Ubx protein within their CNS. (B) Western blot analysis of late wild-type and *elav^5^* embryos shows that the spectrum of Ubx protein isoforms produced in wild-type and mutant embryos closely matches the changes observed at the level of mRNA (Fig. 1) and reveals that *elav^5^* embryos express overall lower levels of Ubx protein than their wild-type counterparts. *n*=9 per genotype; error bars indicate s.e.m.; **P<0.01 [Wilcoxon matched-pairs signed rank test (non-parametric *t*-test)]. (C) Profile quantification of Ubx protein expression along the AP axis (axial expression) as shown in A (standard error is indicated by grey shading). (D) Total *Ubx* mRNA levels as detected by amplification of the constant *Ubx* 5′ exon region are significantly reduced in *elav^5^* embryos compared with wild type. *n*=6 per genotype; error bars indicate s.e.m.; *P<0.05 [Wilcoxon matched-pairs signed rank test (non-parametric *t*-test)]. (E) Analysis of *Ubx* transcriptional inputs in *elav* mutants and wild-type embryos using the *35UZ Ubx-lacZ* promoter fusion. No appreciable difference in *Ubx-lacZ* expression is detected between wild-type and *elav^5^* embryos. (F) *Ubx* nascent transcript expression detected by FISH using *Ubx* intronic probes (intron 3) indicates that *elav* mutant embryos show an overall higher signal in *Ubx* nascent transcript foci. Inset shows *Ubx* signal detected in two discrete nuclear foci (orange arrowheads) per nucleus (blue, DAPI). (G) Quantification of *Ubx* nascent transcript expression in wild type and *elav^5^* mutants shows that the distributions of *Ubx* nascent transcript foci [log(10)] versus voxel signal intensity are substantially different among genotypes. Best fit curves are shown. Plotting either the relative number of *Ubx* foci (middle) or relative intensity of *Ubx* foci (right) detected in wild-type and *elav* mutant embryos further confirms that *elav^5^* mutants show an overall higher level of expression of *Ubx* nascent transcripts. *n*=7 per genotype; error bars indicate s.e.m.; *P<0.05 [Wilcoxon matched-pairs signed rank test (non-parametric *t*-test)].

To investigate the biological consequences of ELAV interactions with *Ubx* during development we first sought to explore whether these had an impact on *Ubx* expression. First, we examined the extent to which the effects observed at the level of RNA processing (Fig. 1C-H) were reflected at the protein level. Western blot analysis of whole-embryo protein extracts produced from wild type and *elav^5^* mutants (stage 16) revealed that *elav^5^* mutant embryos produce a larger amount of *Ubx* Ia and reduced levels of *Ubx* IVa compared with wild-type embryos, closely matching the ELAV-dependent changes observed in *Ubx* mRNA isoforms (Fig. 1C-H and Fig. 3B). Interestingly, our western blot experiments also showed that *elav^5^* mutants produced reduced levels of Ubx protein. Given that at this point in development *Ubx* is also expressed in other tissues (e.g. epidermis) we carried out a series of immunostainings to detect Ubx protein expression exclusively in dissected ventral nerve cords (Fig. 3A). These experiments showed that *elav^5^* embryos produce significantly less Ubx protein than their wild-type counterparts at stage 16 (Fig. 3A,C). Heterozygous *elav* mutant embryos showed an intermediate level of expression between homozygous mutants and wild-type embryos (not shown). These data confirmed that ELAV removal leads to a significant reduction in Ubx protein expression.

In principle, ELAV could exert its effects on protein expression at multiple points, including *Ubx* mRNA synthesis, stability and/or the translational process itself (Simone and Keene, 2013). Two observations suggested that ELAV might be affecting *Ubx* RNA processing during the transcription cycle. First, our data demonstrated that ELAV is able to modify two aspects of *Ubx* RNA processing, i.e. AS and APA, both of which occur co-transcriptionally (Di Giammartino et al., 2011; Licatalosi and Darnell, 2010; Proudfoot, 2011). Second, our protein-RNA binding experiments (Fig. 2) indicate that ELAV binds *Ubx* RNAs via interactions with elements present in *Ubx* introns (see above), making it somewhat unlikely that ELAV would remain physically associated to the fully processed *Ubx* mRNA and act directly on the translational process. Based on these considerations, we decided to test the hypothesis that ELAV exerts its effects on Ubx protein expression by affecting *Ubx* transcriptional rates, as this is a regulatory process known to affect the kinetics and outcome of both AS and APA reactions (de la Mata et al., 2003; Licatalosi and Darnell, 2010; Proudfoot, 2011).

To explore this possibility we first tested the total levels of *Ubx* mRNAs produced in wild type and *elav^5^* mutants and observed that, indeed, *elav* mutant embryos show reduced levels of *Ubx* mRNA (Fig. 3D). This finding led us to look more closely at the levels of *Ubx* transcription in *elav* mutants. We first explored the transcriptional activity of *Ubx cis*-regulatory regions using the *Ubx* transcriptional reporter 35UZ (Irvine et al., 1991). This reporter construct includes 35 kb of *Ubx* regulatory DNA linked to the *lacZ* reporter and drives *Ubx*-like *lacZ* expression during embryogenesis (Irvine et al., 1991). Contrary to our expectations, these experiments showed no significant differences in *Ubx* transcriptional activity in the presence and absence of ELAV (Fig. 3E), indicating that ELAV effects on *Ubx* expression were likely to occur after transcriptional initiation. Bearing this in mind, we then looked at the levels of *Ubx* nascent transcripts in wild-type and *elav^5^* embryos, developing a series of fluorescence *in situ* hybridisation (FISH) experiments using intronic probes so as to detect precursor RNAs rather than mature mRNAs as visualised by standard RNA *in situ* hybridisation (Fig. 3F). Remarkably, we found that in ≥70% of *elav^5^* embryos the abundance of *Ubx* nascent transcripts was significantly higher than in their wild-type counterparts at an identical developmental stage (Fig. 3F,G). Furthermore, applying an image segmentation and quantification pipeline based on the Fiji imaging platform (Schindelin et al., 2012) to nascent *Ubx* transcript signals detected in *elav^5^* and wild-type samples, we observed that *elav^5^* embryos show both a higher number of transcriptional foci (Fig. 3G, middle) and an overall higher signal intensity level per focus (Fig. 3G, right).

Based on these experiments, we propose that the absence of ELAV leads to inefficient *Ubx* RNA processing and retention of RNA at the site of transcription, with a consequential reduction in *Ubx* mRNA release from DNA and lower Ubx protein formation, suggesting that ELAV-dependent *Ubx* RNA processing could ‘fine-tune’ the levels of expression of *Ubx* within the nervous system. This model is consistent with our data and with previous reports on other systems (including human beta-globin, glyceraldehyde 3-phosphate dehydrogenase, ribosomal protein L3, DNA damage-inducible transcript 3) indicating that non-canonical RNA processing reactions usually prevent mRNA flow to the cytoplasm by tethering processed mRNAs to DNA around transcription sites (Custodio et al., 1999, 2007; Schwartz et al., 2012).

### ELAV removal leads to respecification of neural differentiation programmes under Ubx control

**Fig. 4.**
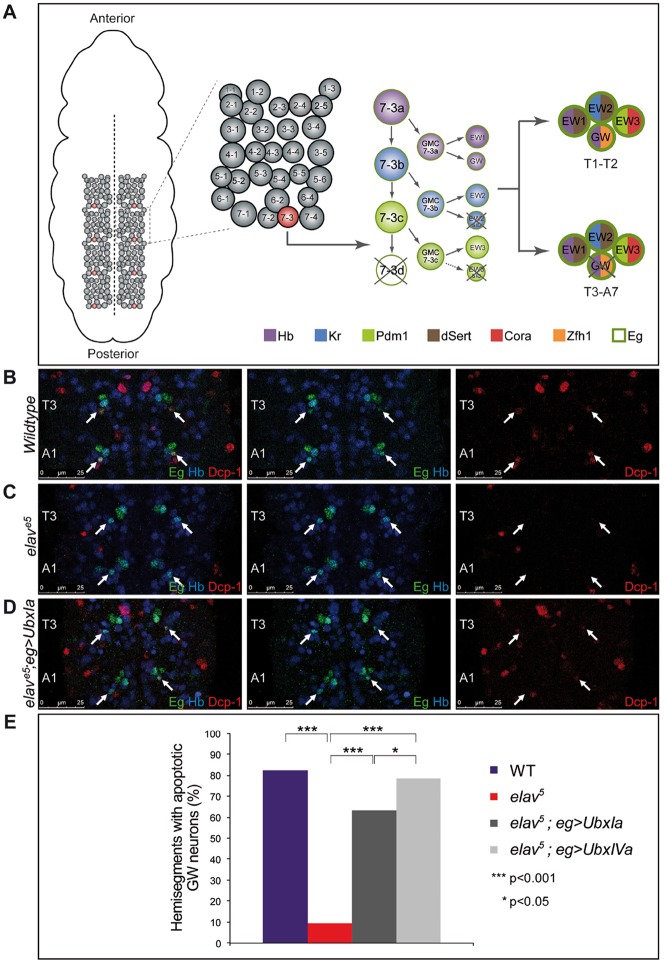
**ELAV regulation leads to changes in neural subroutines under Ubx control.** (A) The *Drosophila* embryonic CNS is formed by a modular segmental array of neuroblasts (left: ventral view of an early embryo, dashed line marks midline; middle left: hemi-segmental set of neuroblasts). Each neuroblast produces a specific cell lineage exhibiting a stereotyped pattern of differentiation. Neuroblast NB7-3 gives rise to six postmitotic progeny cells of which two undergo programmed cell death (crossed out) at early stages and four differentiate into specific neuronal cell types including GW (middle right; see colour code for molecular markers at bottom). In the late embryo, GW undergoes programmed cell death only in segments T3-A7 (see right). (B-D) Confocal imaging of the NB7-3 cluster in stage 16 embryos reveals that in wild-type conditions (B) GW neurons undergo apoptosis specifically in the posterior thorax (T3) and abdomen (A1-A7) (Dcp-1-positive cells in segments T3 and A1, arrows). (C) Notably, genetic removal of *elav* leads to a significant reduction of GW apoptosis. (D) Artificial supply of Ubx protein in *elav^5^* mutants restores normal apoptosis of GW neurons in T3/A1-A7. (E) Quantification of apoptotic behaviour of the GW neuron confirms that reduction of ELAV expression leads to a marked decrease in GW apoptosis [82.03% GW apoptosis in wild type (*n*=128) versus 9.30% in *elav^5^* (*n*=86)]; notably, restoring Ubx protein expression in the system leads to rescue of the apoptotic behaviour of GW. Interestingly, isoforms Ia and IVa have significantly distinct (*P*<0.05, chi-squared test) abilities to restore apoptosis [63.30% (*n*=109) versus 78.30% (*n*=106) GW apoptosis, respectively)], suggesting that *Ubx* AS might play a differential role in the specification of GW apoptosis along the body axis.

Having established the roles of ELAV in Ubx expression within the CNS, we then explored further the biological consequences of ELAV-regulated Hox expression during nervous system development, focusing on the functions of *Ubx* during the establishment of specific cellular programmes within the developing CNS. We reasoned that if ELAV regulation of *Ubx* RNA processing and protein expression were relevant to the biological functions of Ubx during neural development then we should be able to find specific cellular processes affected in *elav* mutants and these should be reverted by artificial provision of Ubx protein. To test these predictions we used a cellular system that had been shown to be sensitive to Ubx dosage: the lineage NB7-3 (Fig. 4A). Here, variations in *Ubx* expression lead to a notable respecification of the apoptosis patterns of the NB7-3–derived GW neuron (Rogulja-Ortmann et al., 2008). In particular, reductions in *Ubx* expression, such as those observed in *Ubx*^*1*^ heterozygotes, lead to a decrease in the proportion of GW neurons undergoing apoptosis in the posterior thorax and abdomen (Rogulja-Ortmann et al., 2008). We examined to what extent the apoptosis patterns in GW neurons were affected in *elav* mutants (Fig. 4B-E). Quantification under confocal microscopy of the apoptosis patterns of GW in over 400 neural hemisegments (stage 16) revealed that levels of ELAV strongly influenced the apoptosis patterns of GW: genetic removal of ELAV leads to a significant reduction in the levels of apoptosis of GW in both thoracic and abdominal segments (Fig. 4E), indicating that changes in the ELAV supply can respecify cellular subroutines under Hox gene control within the developing CNS. Similar effects on GW apoptosis were observed in *elav^ts^* mutants (in which expression of ELAV is reduced but not entirely abolished), which show a clear reduction in GW apoptosis when compared with wild-type embryos (supplementary material Fig. S1). In addition, *elav^ts^* mutants display reduced levels of Ubx protein expression in the CNS, confirming by means of an independent genetic context to the *elav^5^* mutation that reduction in ELAV supply determines a reduction in Ubx protein expression (supplementary material Fig. S5).

Furthermore, the observed reduction in Ubx protein expression level in *elav^5^* mutants (Fig. 3) is fully consistent with the apoptotic effects observed in NB7-3 in that a reduction of *Ubx* expression is expected to reduce the level of GW apoptosis, exactly as observed (Fig. 4B-E). Remarkably, when we restored expression of *Ubx* mRNAs within the NB7-3 lineage in *elav^5^* embryos we observed normal apoptosis levels of GW (Fig. 4D,E), demonstrating that the effects of ELAV on the cellular behaviour of GW are mediated by *Ubx*. Interestingly, selective expression of distinct *Ubx* splicing isoforms (i.e. *Ubx* Ia versus *Ubx* IVa) in the *elav* mutant background revealed isoform-specific roles of Ubx proteins in the rescue of GW apoptosis patterns: *Ubx* Ia was significantly less efficient than *Ubx* IVa in rescuing GW apoptosis. These observations suggest that individual *Ubx* isoforms might play differential roles in neural differentiation along the AP axis. In addition, the fact that Ubx isoform Ia is less efficient than IVa in rescuing normal GW apoptosis patterns provides another explanation for why GW apoptosis is diminished in *elav^5^* mutants: in the absence of ELAV the system produces overall reduced levels of Ubx protein (Fig. 3A,C) and, in addition, much of the protein formed is of the ‘wrong’ kind (i.e. high Ubx Ia low Ubx IVa) to efficiently activate GW apoptosis. Interestingly, similar effects on apoptosis were observed when studying the role of ELAV in the apoptotic behaviour of MNa neurons in the posterior thoracic segment (T3) (supplementary material Fig. S4), which showed a clear reduction in apoptosis compared with wild-type embryos. These cells are derived from the NB2-4 lineage and their apoptosis profiles were previously shown to rely on Ubx protein level (Rogulja-Ortmann et al., 2008). All in all, our analysis here reveals that ELAV modifies the outcome of specific cellular subroutines under Hox control within the *Drosophila* developing embryonic CNS.

### Artificial expression of ELAV promotes Ubx protein expression in glial cells

**Fig. 5.**
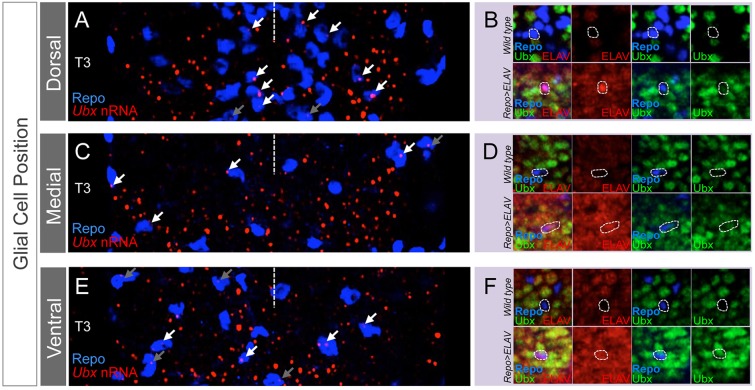
**Artificial expression of ELAV promotes Ubx protein expression in glial cells*.*** (A,C,E) Expression of *Ubx* nascent RNAs (nRNA, red) can be detected (white arrows) in ∼30% of all glial cells (Repo, blue) across dorsal (top), medial (middle) and ventral (bottom) planes within T3 and abdominal segments (not shown). Taking into account that Ubx protein expression is not seen in the glia (Miguel-Aliaga and Thor, 2004), these observations suggest that Ubx protein expression is somehow inhibited in glial cells in which *Ubx* transcription is active. Grey arrows indicate *Ubx* transcribing cells in posterior or anterior segments to T3. Approximate midline positions are indicated by a dashed line; please note that symmetry may not be apparent in these images due to the thinness of confocal imaging. (B,D,F) Artificial expression of ELAV within the glial domain by means of *repo-gal4* leads to an increase in Ubx protein expression in *Ubx* transcribing cells (circled with dashed white lines) in the dorsal (B), medial (D) and ventral (F) glia suggesting that ELAV is sufficient to stabilise Ubx protein expression in those glial cells in which *Ubx* transcription is normally active.

To further explore the biological roles of ELAV in Hox expression within neural tissue we considered the possibility that the absence of ELAV from specific neural cell types (e.g. glial cells) could be related or even contribute to the known lack of Hox protein expression in such cells (Miguel-Aliaga and Thor, 2004). Indeed, the fact that both ELAV and Hox genes are not expressed in the glia could form the basis for an experimental approach to test this idea. Based on the molecular functions of ELAV as an RBP primarily involved in RNA processing and stability, and the fact that its genetic removal does not affect *Ubx* transcription (Fig. 3E), we reasoned that if ELAV had any effects on Hox protein expression in the glia then such effects would only be expected in those glial cells in which *Ubx* transcription was normally active. To determine which glial cells (if any) could meet this proviso, we colabelled glial cells (using anti-Repo antibodies) and nascent *Ubx* RNAs (nRNAs) (making use of a combination of intronic *Ubx* probes) in late wild-type embryos (Fig. 5A,C,E). These experiments revealed that the majority of glial cells did not have an active *Ubx* transcriptional programme. Indeed, in the posterior thoracic segment (T3), which is one of the regions with highest expression of *Ubx*, only about one-third of glial cells transcribes *Ubx* nRNAs, making it unlikely that the absence of ELAV from glia could explain the general lack of Hox expression in these cells.

However, detailed analysis of those cells in which *Ubx* nRNAs could be detected did reveal that artificial expression of ELAV via *repo-gal4* could indeed promote the expression of Ubx protein: forced expression of ELAV in dorsal (e.g. longitudinal glia; Fig. 5B), medial (e.g. lateral cell body glia; Fig. 5D) and ventral (e.g. medial ventral subperineurial glia; Fig. 5F) glial cells resulted in the production of Ubx protein, which is normally not formed in these cells. These experiments suggest that absence of ELAV from glial cells might contribute to the lack of Ubx protein expression in the fraction of these cells in which *Ubx* transcription is normally active.

### Effects of ELAV on the expression of other Hox proteins

**Fig. 6.**
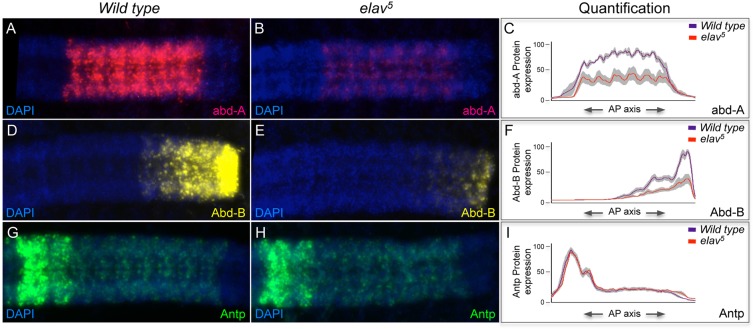
**The effects of ELAV removal on the expression of other Hox proteins than Ubx*.*** (A-C) Comparison of expression levels of Abd-A protein in dissected ventral nerve cords of wild type (A) and *elav^5^* mutants (B) reveals that ELAV removal decreases the overall expression of Abd-A protein within the embryonic CNS. (C) Average profile quantification of Abd-A protein along the AP axis of wild-type and *elav^5^* embryos. (D-F) Expression levels of Abd-B protein are much higher in wild-type (D) than in *elav^5^* mutant (E) embryos, revealing that ELAV removal exerts similar effects across all protein-coding genes within the BX-C. (F) Quantification of Abd-B protein expression in wild-type and *elav^5^* mutant embryos. (G-I) The pattern and expression levels of Antp protein are unaffected by ELAV removal. Antp protein expression levels in wild-type embryos (G) are comparable to those in *elav^5^* mutant embryos (H) indicating that the effects of ELAV on Hox protein expression within the CNS vary from gene to gene. (I) Quantification of Antp protein expression in wild-type and *elav^5^* embryos. (C,F,I) Grey shading represents standard error. (A,B,D,E,G,H) DAPI, blue.

Finally, we decided to investigate the generality of our observations by testing whether ELAV was capable of exerting any effects on the expression of other Hox genes within the nervous system. We looked at the effects of ELAV removal on the other Hox genes within the *Drosophila* Bithorax complex (BX-C), namely *abdominal A* (*abd-A*) and *Abdominal B* (*Abd-B*) (Sánchez-Herrero et al., 1985), given that these genes also display neural-specific patterns of RNA processing (Thomsen et al., 2010). Immunostaining of late embryos (late stage 16) using anti-Abd-A and anti-Abd-B antibodies (Fig. 6A,B,D,E) established that expression of both Abd-A and Abd-B proteins was markedly reduced when ELAV was genetically removed, demonstrating that our observations concerning the effects of ELAV on Ubx expression reflected a more general case concerning all Hox proteins encoded within the BX-C. Nonetheless, the reduction in Ubx, Abd-A and Abd-B protein expression in the absence of ELAV could also be explained by a potentially pleiotropic effect of ELAV on neural gene expression: if ELAV were required for the normal expression and function of cellular components involved in gene expression (e.g. ribosomal proteins) then its removal would be expected to impact the expression of a large number of genes, including the Hox genes. Further experiments made this possibility very unlikely, as the expression of another Hox gene, *Antennapedia* (*Antp*), is unaffected by ELAV removal (Fig. 6G-I), demonstrating that the effects of ELAV on BX-C genes are specific and not the consequence of a general shutdown of protein synthesis in neural tissue caused by the absence of ELAV.

## DISCUSSION

Our work shows that the pan-neural RBP ELAV regulates *Ubx* RNA processing, expression and function within the *Drosophila* embryonic CNS, demonstrating that changes in ELAV level respecify cellular subroutines under *Ubx* control. Based on these findings we propose a model whereby the regulation of Hox gene expression by RNA processing factors adapts the morphogenesis of the nervous system according to axial level by specifically activating local programmes of cell differentiation.

Our study also adds to the understanding of the biological roles of ELAV/Hu by revealing that ELAV is part of the molecular machinery underlying an intrinsic and highly specific programme of cell death within the developing *Drosophila* CNS. This is, to our knowledge, the first demonstration of a specific cellular role for this neural protein. Based on the recent finding that ELAV controls the formation of 3′UTR extensions of several other *Drosophila* mRNAs (Hilgers et al., 2012), we envisage that our present findings are likely to have revealed the first of a wider set of cellular functions played by ELAV through the regulation of its targets in neural tissue.

The notion that the activation of particular patterns of Hox RNA processing via RBPs such as ELAV/Hu can lead to significant fluctuations in Hox protein expression in selected cellular environments suggests the existence of a novel regulatory framework whereby cellular decision making within neural tissue could be adapted to axial level via specific RNA processing programmes triggered and controlled by RNA regulatory factors.

At the molecular level, we interpret the changes observed in *Ubx* RNA processing patterns in the absence of ELAV to represent the outcome of those transcription/processing rounds that were completed despite the absence of this crucial regulator of the process. According to this view, the unavailability of ELAV, with the consequential effects on normal *Ubx* RNA processing, leads to the retention and accumulation of *Ubx* RNAs close to the site of transcription. This interpretation is in line with previous work suggesting that disruption of normal RNA processing reactions can prevent mRNA flow to the cytoplasm by tethering of incompletely processed RNAs to DNA around sites of transcription (Custodio et al., 1999, 2007; Schwartz et al., 2012). We speculate that the cell must have ways to determine, via a series of molecular devices, whether a certain RNA processing pathway has been completed successfully and, should this be not apparent, to trigger an appropriate course of action that seeks to prevent potentially deleterious RNAs from proceeding to export and translation.

Finally, taking into consideration that: (1) Hox genes are evolutionarily conserved between insects and mammals; (2) mammalian Hox genes are key developmental regulators of neural differentiation and undergo substantial levels of RNA processing (P.P. and C.R.A., unpublished); (3) ELAV/Hu RBPs are also evolutionarily conserved between insects and mammals (Yao et al., 1993); and (4) mutations in ELAV-like Hu proteins lead to various forms of neural pathology in humans, we envisage that our findings in *Drosophila* might be of general relevance for understanding the molecular and cellular specification of Hox gene function during the development of the mammalian nervous system. We are currently testing this possibility in the mouse, in which the role of Hox genes during neural specification, differentiation and connectivity has been studied in great detail (Phillippidou and Dasen, 2013).

## MATERIALS AND METHODS

### Fly strains

Flies were cultured following standard procedures at 25°C on a 12 h light/dark cycle. Oregon Red was used as a wild-type strain. The following mutant fly strains were used: *elav^5^* null mutant (Robinow and White, 1991) and *UAS-Elav^2e2^*, *UAS-Elav^3e1^* (both kindly provided by Matthias Soller, University of Birmingham, UK), *UAS-Apollinaire* (Bardet et al., 2008), *elav^ts1^* (Bloomington Stock Center), *Act5c-GAL4* (a gift from Rob Ray, University of Sussex, UK), *Ubx-35UZ* (Irvine et al., 1991), *eagle-Gal4* (MZ360) (Dittrich et al., 1997), *UAS-UbxIa* and *UAS-UbxIVa* (Reed et al., 2010).

### Embryo collection, RNA isolation and RT-PCR

Embryos were collected using standard procedures. For *in situ* hybridisations and antibody stainings, embryos were fixed following standard procedures. For RT-PCR, total RNA was extracted from staged embryo collections using TRI Reagent (Sigma), followed by RNase-free DNaseI treatment (New England BioLabs). Total RNA (1-2 μg) was used for cDNA synthesis using random hexamer or oligo(dT) primers and MuLV reverse transcriptase (Invitrogen). Expression values were normalised using *RpL32* (*Rp49*). At least three independent biological replicates were performed.

### RNA *in situ* hybridisations

Embryos were fixed using standard protocols. Templates of RNA probes for RNA *in situ* hybridisation were obtained from PCR-amplified genomic fragments cloned into pGEM-T (Promega). RNA probes were labelled using a digoxigenin (DIG) RNA Labelling Kit (SP6/T7; Roche) according to the manufacturer's instructions. Embryos were treated as described (Beckervordersandforth et al., 2008) and hybridised with riboprobes according to standard protocols. RNA probes were detected using anti-DIG-AP (Roche; 1:2000) and a chromogenic reaction using NBT/BCIP substrate (Roche). Enzymatic detection reactions with NBT/BCIP (Roche) were carried out in parallel and stopped at exactly the same time for probes targeting universal and distal 3′UTR sequences to ensure comparability of results. Fluorescent detection of RNA probes was performed using anti-DIG-POD (Roche; 1:300) followed by FITC or Cy3 TSA Plus Amplification Kit (PerkinElmer; 1:50). Subsequent imaging was performed on a Zeiss Axiophot confocal microscope and the images were processed using ImageJ and Adobe Photoshop.

### Immunocytochemistry

Antibody stains were performed following standard procedures. Primary antibodies were monoclonal mouse anti-Ubx (FP3.38, a gift from Robert White, University of Cambridge; 1:20), mouse anti-Antp (4C3; 1:20), mouse anti-Abd-B (1A2E9; 1:20), rat anti-ELAV (7E8410; 1:300) and mouse anti-ELAV (9F8A9; 1:300) (all from Developmental Studies Hybridoma Bank); goat anti-Abd-A (dH-17, Santa Cruz Biotechnology; 1:20); rabbit anti-Eg (Dittrich et al., 1997; 1:500); mouse anti-Eg (a gift from Chris Doe, University of Oregon, USA; 1:100); rabbit cleaved Drosophila Dcp-1 (Asp216, Cell Signaling; 1:50); guinea pig anti-Hb (J. Urban, University of Mainz, Germany; 1:500), rabbit anti-β-gal (A11132, Molecular Probes; 1:300), rabbit anti-GFP (A6455, Molecular Probes; 1:300) and rabbit anti-Repo (as described by Halter et al., 1995; 1:500). Secondary antibodies used were anti-mouse-A488 (A21202; 1:500), anti-rat-A488 (A21202; 1:500) and anti-rabbit-Alexa568 (A10042; 1:500) (all from Molecular Probes); anti-rabbit-Rhodamine (711-025-152; 1:500), anti-rat-Rhodamine (712-026-153; 1:500), anti-guinea pig-Cy5 (706-175-148: 1:500), anti-rat-DyLight 405 (712-475-153; 1:500), anti-goat-Cy3 (705-165-003; 1:500) (all from Jackson ImmunoResearch Laboratories).

### Oligo *in situ* hybridisation

Embryos were fixed in 4% paraformaldehyde (PFA; freshly dissolved in PBS):heptane (1:4) solution for 20 min. Following devitellinisation and methanol washes, embryos were refixed in 4% PFA for 20 min. Permeabilisation was performed in 3 µg/ml Proteinase K solution for 13 min at 22°C, followed by incubation on ice for 1 h. Proteinase K was inactivated by washes in 2 mg/ml glycine in PBTween (PBS with 0.1% Tween 20) and embryos refixed for 20 min in 4% PFA. Prehybridisation was carried out overnight at 37°C. DIG-labelled oligo probes were hybridised for 1 day at 37°C, washed in hybridisation buffer at 40°C and in PBTween at room temperature. For detection of oligo probes, embryos were incubated with sheep anti-DIG-AP antibody (Roche) and stained for 20 h at 4°C using the ABC Kit (Vectastain). After a series of washes, embryos were fixed in 4% formaldehyde. For identification of *elav^5^* mutant embryos, a rabbit anti-ß-gal antibody was used (55976, MP Biomedicals; 1:1000). Embryos were mounted and stored in 70% glycerol.

### Bioinformatic search of ultraconserved ELAV targets

We scanned the *Ubx* sequence of *Drosophila melanogaster* for elements with high similarity to experimentally validated EBSs present in the previously reported ELAV target genes *Neuroglian* (*Nrg*, Nrg-like) (TTTTTGTTGT, TTGTTTTTTT, TTTGTTTTT, TTTTATTTAT, TTTTTTTT) (Lisbin et al., 2001) and *erect wing* (*ewg*, ewg-like) (AAUUUUUU, CAUUUUUU) (Soller and White, 2003). To explore the evolutionary conservation of these elements across related *Drosophila* species we retrieved the sequences corresponding to the full *Ubx* transcription unit from the 12 fully sequenced drosophilids from the UCSC Genome Browser (*D. melanogaster*, *D. simulans*, *D. sechellia*, *D. yakuba*, *D. erecta*, *D. ananassae*, *D. pseudoobscura*, *D. persimilis*, *D. willistoni*, *D. mojavensis*, *D. virilis*, *D. grimshawi*) and confirmed their identity as Ubx-encoding sequences using BLAST. *Ubx* sequences derived from different *Drosophila* species were then aligned using the mVISTA LAGAN algorithm (Brudno et al., 2003). We defined as putative ELAV binding sites (pEBSs) all those elements that: (1) presented an exact match to those present in *Nrg* and *ewg* and (2) were evolutionarily conserved in a homologous position across eight or more of the 12 drosophilid species (i.e. ultraconserved). In addition to sequence elements similar to those present in *Nrg* and *ewg* RNAs, we also included in our list of *Ubx* pEBSs a previously described AU-rich element (ARE) present in the 3′UTR of *D. melanogaster Ubx* (Cairrao et al., 2009) given that AREs were shown to be crucial regulators of RNA metabolism through binding of Hu proteins (López de Silanes et al., 2004). All 16 pEBSs are presented in supplementary material Fig. S3.

Site-specific mutation of the putative ELAV binding sequences contained within EBS3 and EBS8 (site 3 WT, ATTTTTT; site 3 Mut, AGTGTGT; site 8A WT, TTTTTGTTT; site 8A Mut, TGTGTGTTT; site 8B WT, TTTGTTTT; site 8B Mut, TGTGTGTG), as used in EMSA, was carried out by *Dpn*I-mediated site-directed mutagenesis.

### EMSA experiments

Recombinant GST-ELAV protein was produced in *E. coli* using an expression vector kindly provided by Matthias Soller (described in Soller and White, 2003) following the manufacturer's instructions (Amersham). GST tag was cleaved with PreScission Protease (Amersham). EMSA experiments were performed as described by Soller and White (Soller and White, 2003). In brief, gel purified ‘body’ labelled RNA was incubated with tRNA (50 μg/ml) at 65°C for 5 min, renatured at room temperature for 10 min and then mixed with binding buffer (50 mM Tris-HCl pH 7.5, 40 mM KCl, 35 mM NaCl, 25 μg/ml tRNA, 0.5 mM DTT, 50 μg/ml BSA) in a total of 10 μl, and incubated at room temperature for 20 min with either 0, 200, 400 or 600 mM ELAV protein. Ten microlitres reaction with 3 μl 50% glycerol were loaded on 4% (80:1 acrylamide/bisacrylamide) polyacrylamide native gels and run at room temperature at 250 V in 0.5×TBE. Gels were dried and exposed to phosphorimager plates.

### UV cross-linking experiments

Uniformly labelled RNA (typically 700 pM) was incubated in 5× binding buffer (400 mM KCl, 100 mM HEPES pH 7.6, 20 mM MgCl_2_, 25% glycerol and 5 mM DTT), 1 µl tRNA (1 mg/ml) and 38 µg/µl ELAV protein or 40% nuclear extract in 10 µl total volume. The mix was incubated at room temperature for 10 min and then divided into two samples, one of which was subsequently UV cross-linked (Stratalinker) for 12 min on ice whereas the other (negative control) was kept on ice in the absence of UV. Samples were then treated with RNaseA at 37°C for 15 min. Reaction products were resolved by SDS-PAGE and dried gels were exposed to phosphorimager plates.

### Cross-linking and immunoprecipitation (CLIP) experiments

Immunoprecipitation of nuclear extracts from an overnight collection of embryos was performed following the RNA immunoprecipitation protocol of Hilgers et al. (Hilgers et al., 2012), except that the nuclear extracts were incubated overnight with 2 μg mouse anti-ELAV 9F8A9 or mouse anti-Tub E7 antibodies (Developmental Studies Hybridoma Bank).

## Supplementary Material

Supplementary Material

## References

[DEV101519C1] Akam M. E. Martinez-Arias A. (1985). The distribution of Ultrabithorax transcripts in Drosophila embryos. EMBO J. 4, 1689-1700 16453626 10.1002/j.1460-2075.1985.tb03838.x PMC554405

[DEV101519C2] Alonso C. R. (2002). Hox proteins: sculpting body parts by activating localized cell death. Curr. Biol. 12, R776-R778 10.1016/S0960-9822(02)01291-5 12445403

[DEV101519C3] Alonso C. R. (2012). A complex ‘mRNA degradation code’ controls gene expression during animal development. Trends Genet. 28, 78-88 10.1016/j.tig.2011.10.005 22257633

[DEV101519C4] Alonso C. R. Akam M. (2003). A Hox gene mutation that triggers nonsense-mediated RNA decay and affects alternative splicing during Drosophila development. Nucleic Acids Res. 31, 3873-3880 10.1093/nar/gkg482 12853602 PMC167643

[DEV101519C5] Alonso C. R. Wilkins A. S. (2005). The molecular elements that underlie developmental evolution. Nat. Rev. Genet. 6, 709-715 10.1038/nrg1676 16094311

[DEV101519C6] Arendt D. Nubler-Jung K. (1999). Comparison of early nerve cord development in insects and vertebrates. Development 126, 2309-2325 10225991 10.1242/dev.126.11.2309

[DEV101519C7] Artero R. D. Akam M. Pérez-Alonso M. (1992). Oligonucleotide probes detect splicing variants in situ in Drosophila embryos. Nucleic Acids Res. 20, 5687-5690 10.1093/nar/20.21.5687 1454531 PMC334403

[DEV101519C8] Bardet P.-L. Kolahgar G. Mynett A. Miguel-Aliaga I. Briscoe J. Meier P. Vincent J.-P. (2008). A fluorescent reporter of caspase activity for live imaging. Proc. Natl. Acad. Sci. U.S.A. 105, 13901-13905 10.1073/pnas.0806983105 18779587 PMC2544551

[DEV101519C9] Beckervordersandforth R. M. Rickert C. Altenhein B. Technau G. M. (2008). Subtypes of glial cells in the Drosophila embryonic ventral nerve cord as related to lineage and gene expression. Mech. Dev. 125, 542-557 10.1016/j.mod.2007.12.004 18296030

[DEV101519C10] Bomze H. M. Lopez A. J. (1994). Evolutionary conservation of the structure and expression of alternatively spliced Ultrabithorax isoforms from Drosophila. Genetics 136, 965-977 7911773 10.1093/genetics/136.3.965 PMC1205900

[DEV101519C11] Brudno M. Do C. B. Cooper G. M. Kim M. F. Davydov E.; NISC Comparative Sequencing Program, Green E. D. Sidow A. Batzoglou S. (2003). LAGAN and Multi-LAGAN: efficient tools for large-scale multiple alignment of genomic DNA. Genome Res. 13, 721-731 10.1101/gr.926603 12654723 PMC430158

[DEV101519C12] Cairrao F. Halees A. S. Khabar K. S. A. Morello D. Vanzo N. (2009). AU-rich elements regulate Drosophila gene expression. Mol. Cell. Biol. 29, 2636-2643 10.1128/MCB.01506-08 19273595 PMC2682044

[DEV101519C13] Custodio N. Carmo-Fonseca M. Geraghty F. Pereira H. S. Grosveld F. Antoniou M. (1999). Inefficient processing impairs release of RNA from the site of transcription. EMBO J. 18, 2855-2866 10.1093/emboj/18.10.2855 10329631 PMC1171366

[DEV101519C14] Custodio N. Vivo M. Antoniou M. Carmo-Fonseca M. (2007). Splicing- and cleavage-independent requirement of RNA polymerase II CTD for mRNA release from the transcription site. J. Cell Biol. 179, 199-207 10.1083/jcb.200612109 17938247 PMC2064756

[DEV101519C15] de la Mata M. Alonso C. R. Kadener S. Fededa J. P. Blaustein M. Pelisch F. Cramer P. Bentley D. Kornblihtt A. R. (2003). A slow RNA polymerase II affects alternative splicing in vivo. Mol. Cell 12, 525-532 10.1016/j.molcel.2003.08.001 14536091

[DEV101519C16] de Navas L. F. Reed H. Akam M. Barrio R. Alonso C. R. Sanchez-Herrero E. (2011). Integration of RNA processing and expression level control modulates the function of the Drosophila Hox gene Ultrabithorax during adult development. Development 138, 107-116 10.1242/dev.051409 21115609

[DEV101519C17] Di Giammartino D. C. Nishida K. Manley J. L. (2011). Mechanisms and consequences of alternative polyadenylation. Mol. Cell 43, 853-866 10.1016/j.molcel.2011.08.017 21925375 PMC3194005

[DEV101519C18] Dittrich R. Bossing T. Gould A. P. Technau G. M. Urban J. (1997). The differentiation of the serotonergic neurons in the Drosophila ventral nerve cord depends on the combined function of the zinc finger proteins Eagle and Huckebein. Development 124, 2515-2525 9216994 10.1242/dev.124.13.2515

[DEV101519C19] Good P. J. (1995). A conserved family of elav-like genes in vertebrates. Proc. Natl. Acad. Sci. U.S.A. 92, 4557-4561 10.1073/pnas.92.10.4557 7753842 PMC41983

[DEV101519C20] Halter D. A. Urban J. Rickert C. Ner S. S. Ito K. Travers A. A. Technau G. M. (1995). The homeobox gene *repo* is required for the differentiation and maintenance of glia function in the embryonic nervous system of Drosophila melanogaster. Development 121, 317-332 7768175 10.1242/dev.121.2.317

[DEV101519C21] Hatton A. R. Subramaniam V. Lopez A. J. (1998). Generation of alternative Ultrabithorax isoforms and stepwise removal of a large intron by resplicing at exon-exon junctions. Mol. Cell 2, 787-796 10.1016/S1097-2765(00)80293-2 9885566

[DEV101519C22] Hilgers V. Lemke S. B. Levine M. (2012). ELAV mediates 3′ UTR extension in the Drosophila nervous system. Genes Dev. 26, 2259-2264 10.1101/gad.199653.112 23019123 PMC3475798

[DEV101519C23] Irvine K. D. Helfand S. L. Hogness D. S. (1991). The large upstream control region of the Drosophila homeotic gene Ultrabithorax. Development 111, 407-424 1680046 10.1242/dev.111.2.407

[DEV101519C24] Kornfeld K. Saint R. B. Beachy P. A. Harte P. J. Peattie D. A. Hogness D. S. (1989). Structure and expression of a family of Ultrabithorax mRNAs generated by alternative splicing and polyadenylation in Drosophila. Genes Dev. 3, 243-258 10.1101/gad.3.2.243 2565858

[DEV101519C25] Koushika S. P. Soller M. White K. (2000). The neuron-enriched splicing pattern of Drosophila erect wing is dependent on the presence of ELAV protein. Mol. Cell. Biol. 20, 1836-1845 10.1128/MCB.20.5.1836-1845.2000 10669758 PMC85364

[DEV101519C26] Licatalosi D. D. Darnell R. B. (2010). RNA processing and its regulation: global insights into biological networks. Nat. Rev. Genet. 11, 75-87 10.1038/nrg2673 20019688 PMC3229837

[DEV101519C27] Lisbin M. J. Qiu J. White K. (2001). The neuron-specific RNA-binding protein ELAV regulates neuroglian alternative splicing in neurons and binds directly to its pre-mRNA. Genes Dev. 15, 2546-2561 10.1101/gad.903101 11581160 PMC312793

[DEV101519C28] Lopez A. J. Hogness D. S. (1991). Immunochemical dissection of the Ultrabithorax homeoprotein family in Drosophila melanogaster. Proc. Natl. Acad. Sci. U.S.A. 88, 9924-9928 10.1073/pnas.88.22.9924 1719557 PMC52839

[DEV101519C29] López de Silanes I. Zhan M. Lal A. Yang X. Gorospe M. (2004). Identification of a target RNA motif for RNA-binding protein HuR. Proc. Natl. Acad. Sci. U.S.A. 101, 2987-2992 10.1073/pnas.0306453101 14981256 PMC365732

[DEV101519C30] Lumsden A. Keynes R. (1989). Segmental patterns of neuronal development in the chick hindbrain. Nature 337, 424-428 10.1038/337424a0 2644541

[DEV101519C31] Lumsden A. Krumlauf R. (1996). Patterning the vertebrate neuraxis. Science 274, 1109-1115 10.1126/science.274.5290.1109 8895453

[DEV101519C58] Mallo M. Alonso C. R. (2013). The regulation of Hox gene expression during animal development. Development 140, 3951-3963 10.1242/dev.068346 24046316

[DEV101519C32] Mann R. S. Hogness D. S. (1990). Functional dissection of Ultrabithorax proteins in D. melanogaster. Cell 60, 597-610 10.1016/0092-8674(90)90663-Y 2105847

[DEV101519C33] McGinnis W. Krumlauf R. (1992). Homeobox genes and axial patterning. Cell 68, 283-302 10.1016/0092-8674(92)90471-N 1346368

[DEV101519C34] Miguel-Aliaga I. Thor S. (2004). Segment-specific prevention of pioneer neuron apoptosis by cell-autonomous, postmitotic Hox gene activity. Development 131, 6093-6105 10.1242/dev.01521 15537690

[DEV101519C35] O'Connor M. B. Binari R. Perkins L. A. Bender W. (1988). Alternative RNA products from the Ultrabithorax domain of the bithorax complex. EMBO J. 7, 435-445 2452731 10.1002/j.1460-2075.1988.tb02831.x PMC454339

[DEV101519C36] Patraquim P. Warnefors M. Alonso C. R. (2011). Evolution of Hox post-transcriptional regulation by alternative polyadenylation and microRNA modulation within 12 Drosophila genomes. Mol. Biol. Evol. 28, 2453-2460 10.1093/molbev/msr073 21436120

[DEV101519C37] Philippidou P. Dasen J. S. (2013). Hox genes: choreographers in neural development, architects of circuit organization. Neuron 80, 12-34 10.1016/j.neuron.2013.09.020 24094100 PMC3835187

[DEV101519C38] Proudfoot N. J. (2011). Ending the message: poly(A) signals then and now. Genes Dev. 25, 1770-1782 10.1101/gad.17268411 21896654 PMC3175714

[DEV101519C39] Reed H. C. Hoare T. Thomsen S. Weaver T. A. White R. A. H. Akam M. Alonso C. R. (2010). Alternative splicing modulates Ubx protein function in Drosophila melanogaster. Genetics 184, 745-758 10.1534/genetics.109.112086 20038634 PMC2845342

[DEV101519C40] Reichert H. (2002). Conserved genetic mechanisms for embryonic brain patterning. Int. J. Dev. Biol. 46, 81-87 11902691

[DEV101519C41] Robinow S. White K. (1988). The locus elav of Drosophila melanogaster is expressed in neurons at all developmental stages. Dev. Biol. 126, 294-303 10.1016/0012-1606(88)90139-X 3127258

[DEV101519C42] Robinow S. White K. (1991). Characterization and spatial distribution of the ELAV protein during Drosophila melanogaster development. J. Neurobiol. 22, 443-461 10.1002/neu.480220503 1716300

[DEV101519C43] Robinow S. Campos A. R. Yao K. M. White K. (1988). The elav gene product of Drosophila, required in neurons, has three RNP consensus motifs. Science 242, 1570-1572 10.1126/science.3144044 3144044

[DEV101519C44] Rogulja-Ortmann A. Renner S. Technau G. M. (2008). Antagonistic roles for Ultrabithorax and Antennapedia in regulating segment-specific apoptosis of differentiated motoneurons in the Drosophila embryonic central nervous system. Development 135, 3435-3445 10.1242/dev.023986 18799545

[DEV101519C45] Sánchez-Herrero E. Vernós I. Marco R. Morata G. (1985). Genetic organization of the Drosophila bithorax complex. Nature 313, 108-113 10.1038/313108a0 3917555

[DEV101519C46] Schindelin J. Arganda-Carreras I. Frise E. Kaynig V. Longair M. Pietzsch T. Preibisch S. Rueden C. Saalfeld S. Schmid B. (2012). Fiji: an open-source platform for biological-image analysis. Nat. Methods 9, 676-682 10.1038/nmeth.2019 22743772 PMC3855844

[DEV101519C47] Schwartz J. C. Ebmeier C. C. Podell E. R. Heimiller J. Taatjes D. J. Cech T. R. (2012). FUS binds the CTD of RNA polymerase II and regulates its phosphorylation at Ser2. Genes Dev. 26, 2690-2695 10.1101/gad.204602.112 23249733 PMC3533074

[DEV101519C48] Shimell M. J. Simon J. Bender W. O'Connor M. B. (1994). Enhancer point mutation results in a homeotic transformation in Drosophila. Science 264, 968-971 10.1126/science.7909957 7909957

[DEV101519C49] Simon J. Peifer M. Bender W. O'Connor M. (1990). Regulatory elements of the bithorax complex that control expression along the anterior-posterior axis. EMBO J. 9, 3945-3956 1979031 10.1002/j.1460-2075.1990.tb07615.x PMC552165

[DEV101519C50] Simone L. E. Keene J. D. (2013). Mechanisms coordinating ELAV/Hu mRNA regulons. Curr. Opin. Genet. Dev. 23, 35-43 10.1016/j.gde.2012.12.006 23312841 PMC3617084

[DEV101519C51] Soller M. White K. (2003). ELAV inhibits 3′-end processing to promote neural splicing of ewg pre-mRNA. Genes Dev. 17, 2526-2538 10.1101/gad.1106703 14522950 PMC218147

[DEV101519C52] Soller M. White K. (2005). ELAV multimerizes on conserved AU_4-6_ motifs important for *ewg* splicing regulation. Mol. Cell. Biol. 25, 7580-7591 10.1128/MCB.25.17.7580-7591.2005 16107705 PMC1190278

[DEV101519C53] Subramaniam V. Bomze H. M. Lopez A. J. (1994). Functional differences between Ultrabithorax protein isoforms in Drosophila melanogaster: evidence from elimination, substitution and ectopic expression of specific isoforms. Genetics 136, 979-991 7911774 10.1093/genetics/136.3.979 PMC1205901

[DEV101519C54] Thomsen S. Azzam G. Kaschula R. Williams L. S. Alonso C. R. (2010). Developmental RNA processing of 3′UTRs in Hox mRNAs as a context-dependent mechanism modulating visibility to microRNAs. Development 137, 2951-2960 10.1242/dev.047324 20667912

[DEV101519C55] Thor S. (1995). The genetics of brain development: conserved programs in flies and mice. Neuron 15, 975-977 10.1016/0896-6273(95)90084-5 7576663

[DEV101519C56] Wang X. Tanaka T. M. (2001). Structural basis for recognition of AU-rich element RNA by the HuD protein. Nat. Struct. Biol. 8, 141-145 10.1038/84131 11175903

[DEV101519C57] Yao K. M. Samson M. L. Reeves R. White K. (1993). Gene elav of Drosophila melanogaster: a prototype for neuronal-specific RNA binding protein gene family that is conserved in flies and humans. J. Neurobiol. 24, 723-739 10.1002/neu.480240604 8331337

