# Supplementary material for: The RNA-binding protein ELAV regulates Hox RNA processing, expression and function within the Drosophila nervous system
Source: Development. 2014 May;141(10):2046–56. doi: 10.1242/dev.101519 (PMC4132933; doi:10.1242/dev.101519)
Supplement: Supplementary Material [file supp_141_10_2046__index.html]

The RNA-binding protein ELAV regulates Hox RNA processing, expression and function within the Drosophila nervous system — Supplementary Material 

# The RNA-binding protein ELAV regulates Hox RNA processing, expression and function within the *Drosophila* nervous system

## DEV101519 Supplementary Material

**Files in this Data Supplement:**

- **Supplementary Material**
