# Supplementary material for: The RNA-binding protein ELAV regulates Hox RNA processing, expression and function within the Drosophila nervous system
Source: Development. 2014 May;141(10):2046–56. doi: 10.1242/dev.101519 (PMC4132933; doi:10.1242/dev.101519)
Supplement: Supplementary Material [file supp_141.10.2046_DEV101519.pdf]

Supplementary Figure 1

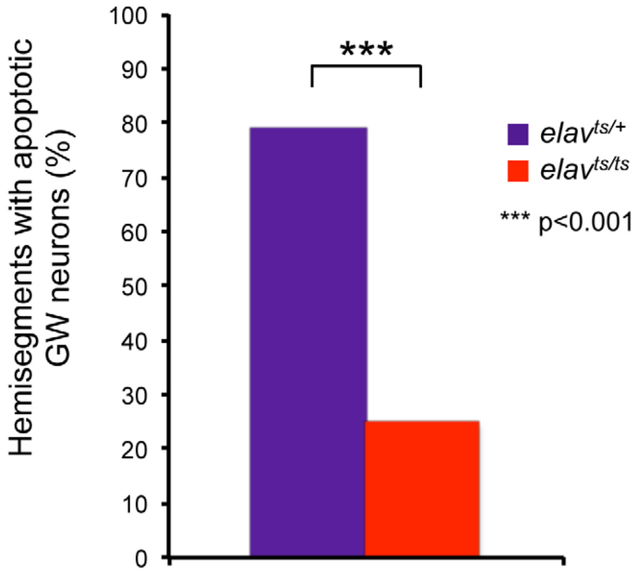

GENOTYPE	GW Apoptosis % (n)
elav$^{ts/+}$	78,7 (47)
elav$^{ts/ts}$	25 (48)

Effects of the *elav*^{ts} allele on the apoptotic behaviour of GW neurons. Analysis of apoptotic behaviour of GW cells in the thoracic/abdominal segments of control *elav*$^{+/ts}$ (purple) and *elav*$^{ts/ts}$ mutants (red) further confirms that genetic removal of ELAV expression leads to a significant change in the incidence of apoptosis on GW neurons.

Supplementary Figure 2

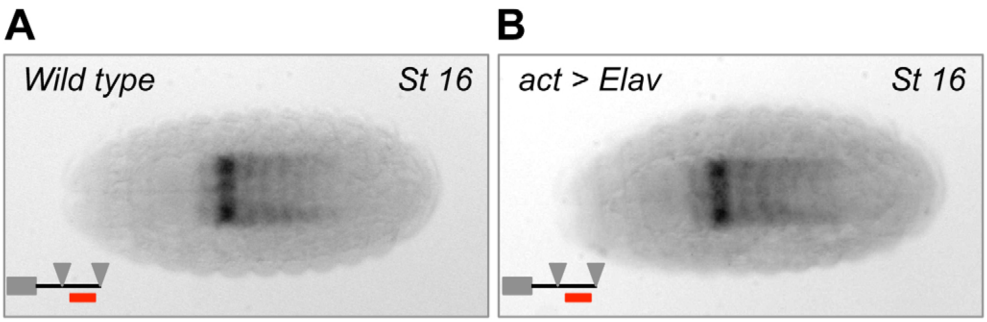

Ectopic expression of ELAV during late embryogenesis does not cause any detectable increase in the expression of long 3'UTR *Ubx* mRNA forms. *RNA in situ* hybridisation of stage 16 embryos with *Ubx* distal 3'UTR probes (symbolised by a red line) shows that the artificial increase of ELAV expression from a UAS-*elav* construct driven by the *actin-gal4* driver (act>Elav, (**B**)) shows no appreciable change in the pattern of long *Ubx* 3'UTR expression when compared to *wild type* embryos (**A**) suggesting that endogenous expression of ELAV at this late embryonic stage is likely saturating and/or that a maximal production of long-3'UTR *Ubx* RNA forms had been achieved.

Supplementary Figure 3

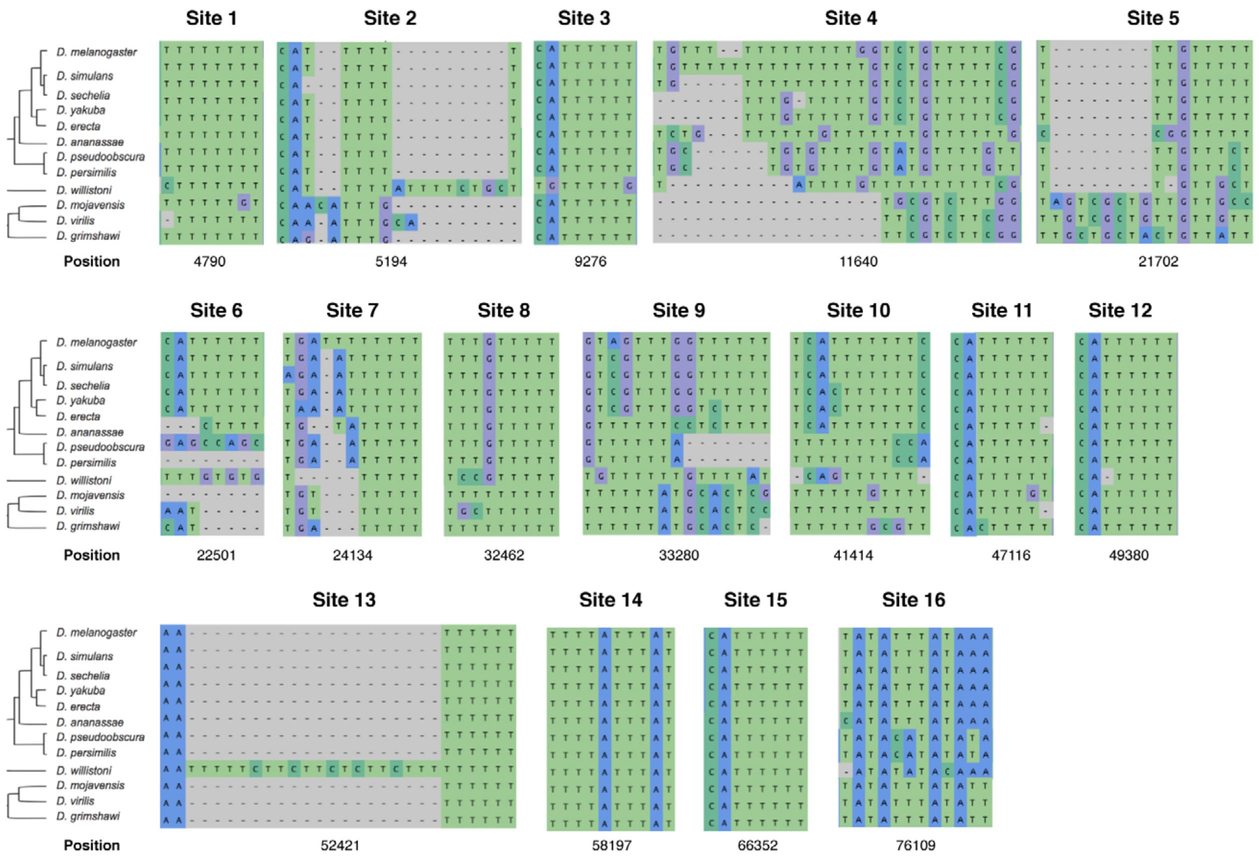

Computational prediction of putative ELAV binding sites (pEBSs) on Ubx RNAs detects sixteen candidate elements. In order to determine putative ELAV binding sites within *Ubx* RNA we combined the scannning of *Drosophila melanogaster Ubx* sequences for elements with high similarity to experimentally validated ELAV-binding sites present in the genes *neuroglian* (*nrg*, Nrg-like) (tttttgttgt, ttgttttttt, tttgttttt, ttttatttat, tttttttt) (Lisbin et al., 2001) and *erect-wing* (*ewg*, ewg-like) (aauuuuuuu, cauuuuuuu) (Soller, M., and K. White, 2003) with an analysis of the evolutionary conservation of these elements across twelve related Drosophila species (*D. melanogaster, D. simulans, D. sechellia, D. yakuba, D. erecta, D. ananassae, D. pseudoobscura, D. persimilis, D. willistoni, D. mojavensis, D. virilis, D. grimshawi*). This approach defined fifteen *putative Elav binding sites* (pEBSs) which (i) presented an exact match to those in *nrg* and *ewg* and (ii) were evolutionarily conserved in a homologous position across eight or more Drosophila species (i.e. ultraconserved) out of the twelve Drosophilid species investigated. In addition to sequence elements similar to those present in *nrg* and *ewg* RNAs we also included in our list of *Ubx* pEBSs a previously described A-U rich element (ARE) present in the 3'UTR of *D. melanogaster Ubx* (Site 16) (Cairrao et al., 2009) given that ARE elements were shown to be critical regulators of RNA metabolism through binding of Hu proteins (Lopez de Silanes et al., 2004).

Supplementary Figure 4

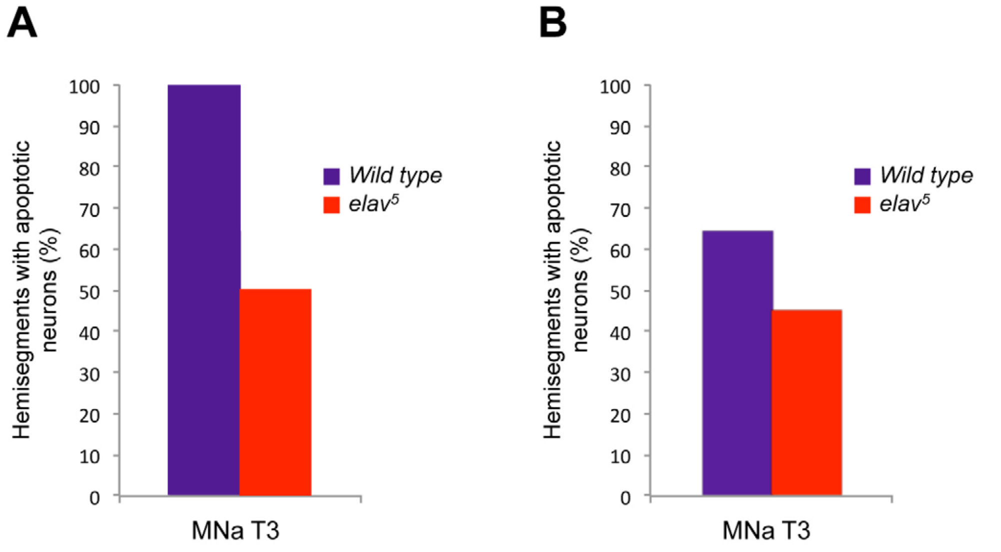

GENOTYPE	MNa T3 Apoptosis % (n)
WT	100 (21)
elav^5	50 (24)

GENOTYPE	MNa T3 Apoptosis % (n)
WT	64,3 (14)
elav^5	45 (20)

Effects of ELAV on the apoptotic behaviour of MNa neurons. Analysis of apoptotic behaviour of MNa cells in the posterior thoracic segment (T3) in wild type (purple) and *elav^5* mutants (red) by means of: (**A**) the fluorescent genetically-encoded caspase sensor *Apollinaire* (Bardet et al. (2008) PNAS 105(37): 13901-13905) and (**B**) the number of Dcp-1$^+$ cells demonstrates that MNa apoptotic rates are markedly reduced (30-50% reduction depending on method) when Elav is absent from the system confirming the same trend observed in the GW motorneuron system.

Supplementary Figure 5

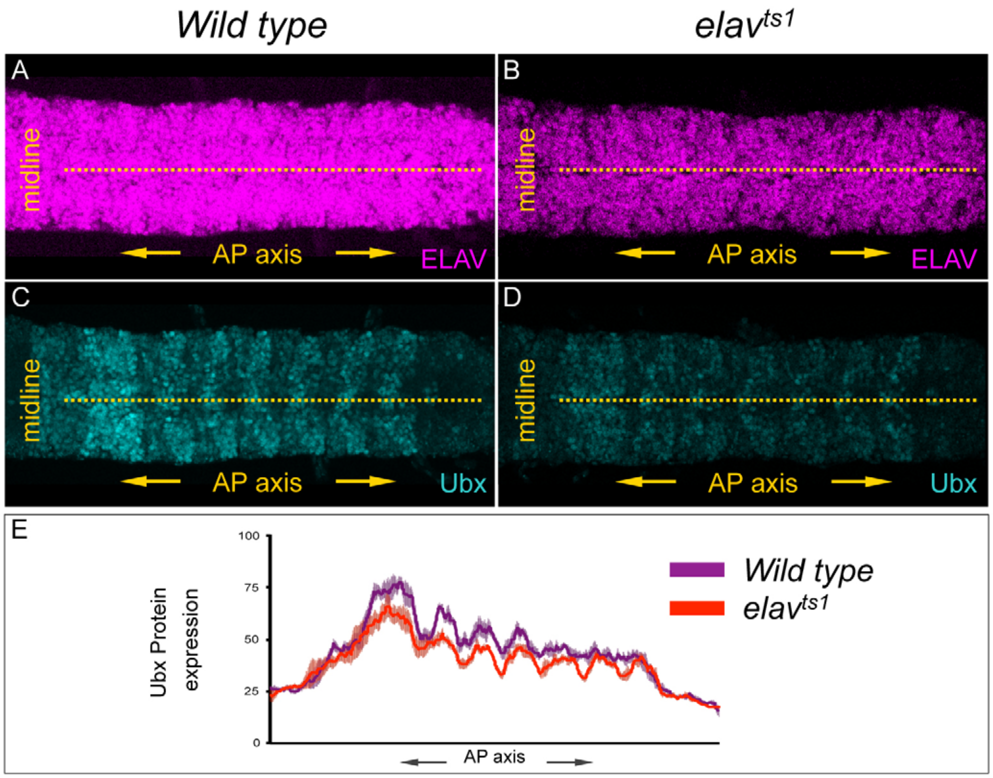

Effects of the *elav^{ts}* allele on expression of Ubx protein. (**A,B**) Comparison of ELAV protein (magenta) expression levels in dissected ventral nerve cords of wild type (A) and *elav^{ts1}* mutant (B) embryos at stage 16 shows that ELAV protein levels are reduced in the *elav^{ts1}* mutant. (**C,D**) Expression levels of Ubx protein (cyan) are clearly higher in wild type (C) than in *elav^{ts1}* mutant embryos (D) further confirming that reducing expression of ELAV protein results in lower levels of Ubx protein within the CNS. (E) Average profile quantification of Ubx protein along the A-P axis of wild type (purple) and *elav^{ts1}* mutant embryos (red) (standard error is indicated in light purple and pink shading, respectively)
